# Validity and reliability of a simplified food frequency questionnaire: a cross sectional study among physical health examination adults in southwest region of China

**DOI:** 10.1186/s12937-020-00630-z

**Published:** 2020-10-06

**Authors:** Zhengyan Cheng, Ping Shuai, Qichuan Qiao, Tingxin Li

**Affiliations:** 1 Department of Pathology, Eastern Hospital, Sichuan Academy of Medical Sciences & Sichuan Provincial People’s Hospital, Chengdu, 610072 China; 2 Health Management Center, Sichuan Academy of Medical Sciences & Sichuan Provincial People’s Hospital, Chengdu, 610072 China; 3 School of Medicine, University of Electronic Science and Technology of China, Chengdu, 611731 China

**Keywords:** Physical health examination, SFFQ, Epidemiological studies, Southwest china, Validity, Reliability

## Abstract

**Background:**

In China, many people are regarded suitable for participating in regular physical examination for diagnosis and prevention of diseases. Some simplified food frequency questionnaires have been designed and used; however, the accuracy of the questionnaire is absent. This study aimed to examine the reliability and validity of simplified food frequency questionnaire (SFFQ) used among adults undergoing physical examination in southwest region of China.

**Methods:**

This was a cross-sectional study conducted among physical health examination adults in the Southwest region of China. A total of 239 participants aged 20–65 were included from February 2019 to June 2019. The performance of SFFQ was evaluated by means of a three-day 24-h dietary record (3R24). The relative validity and agreement was assessed by Pearson’s correlation and intra-class correlation coefficients (*ICC*), respectively.

**Results:**

The median energy-adjusted *ICC* of food groups between SFFQ2 and SFFQ1 was 0.59 (range: 0.49–0.73) and the *ICC* of nutrients was 0.47(range: 0.39–0.76). The Pearson correlation showed a valid comparisons between SFFQ1 and 3R24, ranging from -0.086 to 0.93 for food and 0.21 to 0.71 for nutrition, respectively. The energy-adjustment slightly increased the correlation coefficients.

**Conclusions:**

The reliability and validity of SFFQ was acceptable, and it could be an appropriate dietary assessment tool for the future epidemiological studies conducted among physical health examination adults of southwest China.

Trial registration

CHiCTR, ChiCTR1900020934, Registered 22 January 2019, https://www.chictr.org.cn/edit.aspx?pid=35414&htm=4.

## Introduction

Although quantification and assessment of diet are difficult tasks, they are necessary to check the relationship between nutrients and non-transmissible chronic diseases (NTCDs) [1]. Such studies belonged to the field of nutritional epidemiology.

Several types of instruments are used to assess both present as well as previous diet: the 24 h diet record (R24), food-intake record (FR), and the food frequency questionnaire (FFQ). But all these instruments had their own advantages or limitations. FFQ is a cost-effective method used for dietary assessment in large population groups [2]. Currently, it is widely used in epidemiological studies with the intention to assess the relation between diet and disease. It could provide information on the frequency and proportion of a defined food items list. FFQ is a feasible questionnaire, and involves low cost and low recall bias [3]. It is recommended as one of the first-line methods for dietary measurements, especially when conducting a population survey or community trials. For assessing the factors that have effects on the incidence or prevalence of chronic diseases, the dietary pattern or various food intakes included in FFQ should be valid [4]. Recently, many reports have been put forwarded on the use of FFQ in evaluating single nutrients [5]. Some scholars have reported the use of food frequency questionnaires to assess disease risk [6].

But food intake largely varies based on ethnicity, social, and cultural background of the study population. Validation study should be conducted to improve the application of FFQ within specific samples [7] and accuracy, and reduce bias levels related to disease occurrence [8].

In China, many people are considered suitable for participating in regular physical examination for diagnosis and prevention. It is vital to evaluate the relationship of diet with their physical examination data, which is typically large in size and require continuous collection. This data is an important source for forecasting the risk factors associated with disease. However, in China, the traditional food frequency questionnaires include more than one hundred kinds of foods, which would nearly take 30 min to finish. It is generally assumed that the questionnaire length has a significant effect on the survey response rate as respondents get tired, bored and/or distracted by external factors [5]. Some simplified food frequency questionnaires (SFFQs) have been designed and used in special populations [9, 10]. However, the validity of SFFQ in Chinese population undergoing physical examination is not discussed. It is also unclear as to whether SFFQ could evaluate the intake of samples that are similar to large-scale crowd survey.

Hence, in this study, by combining the traditional FFQ questionnaires of China and the dietary habits of residents of southwest China, a new SFFQ was designed. To collect the dietary intake in the preceding 3 months, its reliability and validity was assessed among adults undergoing physical examination in the Southwest region of China.

## Materials and Methods

### Participants

**Fig. 1:**
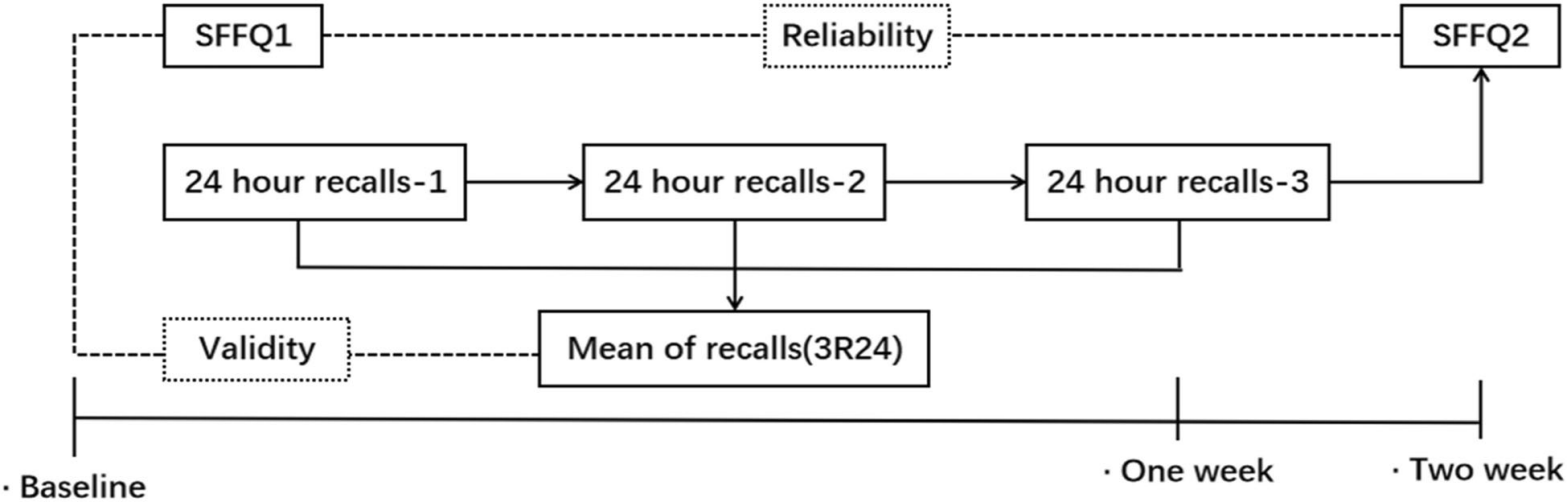
Flow chart of the study for the SFFQ and 3R24

This was a cross-sectional study conducted among adults undergoing physical health examination in southwest region of China. Participants included those undergoing physical examination in the Medical Examination Center of Sichuan Provincial People`s Hospital from February to June in 2019. Subjects aged 20–65, and those who could fill in the questionnaire independently and accepted a phone interview were included. The dietary information was collected using SFFQ and 3R24s. All information was collected by 3 trained interviewers. Participants were included in this study for a period of 2 weeks (Fig. 1). The first SFFQ (SFFQ1) was collected at the medical examination site, and the second SFFQ (SFFQ2) was collected after 2 weeks through phone. The contents of both SFFQ2 and SFFQ1 remained the same, but the order of questions generated by the computer was random. The 3R24s were designed on Saturday, Monday, and Tuesday, in which the two weekdays and one weekend day accounted for intraindividual variation between day types were covered and completed by interviewers through phone. A total of 239 completed 3R24s and SFFQ1 + 2 were collected. All participants were informed about the study and obtained formal consents before being interviewed. This study was approved by the Ethics Committee of the Sichuan Provincial People's Hospital (Approval Number:2017–153), China.

### Dietary Assessment

#### Simplified Food Frequency Questionnaire (SFFQ)

Based on an FFQ used in Chinese population [10], involving the eating habits of southwest China, such as preserved meat, chilli and soy products, a new SFFQ was designed. The SFFQ consisted of 50 items of food. The following three sections were included in the questionnaire: the food list, the frequency response, and the average intake. The food items were divided into 14 categories, Table 1. When filling the SFFQ, all participants were required to indicate how many times they consumed each food category per month/week/day in the past 3 months. The frequency of consumption was measured by selecting one of the following options: (1) never or less than once per month, (2) 1–3 times per month, (3) 1–3 times per week, (4) 4–6 times per week, and (5) how many times per day. Because there was no unified portion size in China, pictures of utensils and food portions were taken into account to assist with the description along with trained investigators. Food intake (g/day) was calculated by multiplying the frequency of consumption by portion size with the mean frequencies of consumption per day for each group.

**Table 1:** Food groups and their components in SFFQ

**Food groups**	**Components**
Cereal	Rice, millet, sorghnm
Bread and noodle	Bread, cake, noodle, flour, pizza
Tubers	Potato, cassava, yam
Red meat	Pork, beef, cow, goat, rabbit
Poultry	Chicken, duck, goose
Aquatic products	Fish, shrimp, aquid, crab
Haslet and processed meat products	Haslet, sausage, ham, preserved meat
Eggs	Boiled eggs, omelette
Dairy	Milk, yaourt, yoghourt, cheese
Vegetables	Leafy greens, chili, vegetables, green beans, vegetable juice
Phytocomycetes	Mushroom, kelp, sea beans
Soy products	Legumes, tofu, soy beans
Fruits	Berries, dried fruits, fruits, fruit juice
Nuts	Cashew nuts, walnuts, peanuts

#### 3-day 24-h Dietary Record (3R24)

The study population completed 3R24s in a week, which included two working days and one rest day. These questionnaires were completed by interviewers through phone over a course of 3-day period. The resulting dietary data included 4,024 “food mentions”, i.e., combination of entire food items of all participants. These data were coded into 14 broad food groups and 12 types of nutrients according to the SFFQ of Chinese Residents Dietary Guidelines’ selection. The mean weights were calculated respectively.

#### Assessment

The daily nutrient consumption of participants was calculated by matching the food code from the China food composition database. If a food was not listed in the database, then the similar food item was used instead of calculating the nutritional values.

### Statistical Analyses

All data were entered twice into the Epidata. Pearson correlation coefficients were used to evaluate the validity of food frequency acquired by SFFQ1 with an average of 3R24 data (food weight). According to a recent systematic review, the validity of FFQs should be re-evaluated because energy, carbohydrate, calcium, and vitamin C intakes were overestimated, especially in females [11. Some scholars' have reported that FFQs tend to have a larger measurement error in underestimating the energy and nutrient intake when compared to 24-h dietary recalls [12]. And the simplified dietary survey questionnaires tend to underestimate the energy and nutrient intake as compared to the longer questionnaires [13]. In our study, after adjusting the energy, 3R24 was taken as reference for SFFQ. The adjusted correlation coefficients were then calculated. The agreement between SFFQ1 and SFFQ2 was assessed by intra-class correlation coefficients (*ICC*) of energy-adjusted food and nutrient intake. All analyses were performed using Statistical Package for the Social Science (SPSS) software, version 17.0 (IBM, Armonk, New York, NY, USA). A P value of <0.05 was considered to be statistically significant.

## Results

### Description of the study population

Approximately 5–8 min were taken to complete one SFFQ under the guidance of investigators. Among the initial 268 participants, 239(89.18%) individuals with a mean age of 44.29(9.12) years have finished both 3R24 and SFFQ1/2. Half of the participants (51.88%) had 3000 + RMB monthly income, but 37.24% of them refused to provide this information. The habit of having breakfast was good, and this accounted for 87% of all participants, which was beyond our expectation. But most of them ate food outside frequently. The characteristics of study population are described in Table 2.

**Table 2:** Characteristics of study subjects

Characteristic	Total(n = 239)	Male(n = 158)	Female(n = 81)
**Age(n,%)**	44.29 (9.12)	45.43(8.45)	42.02(8.23)
< 30	8(3.35)	4(2.53)	4(4.94)
30–40	69(28.87)	43(27.22)	26(32.10)
40–50	100(41.84)	67(42.41)	33(40.74)
50–60	44(18.41)	32(20.25)	12(14.81)
≥ 60	18(7.53)	12(7.59)	6(7.41)
**Education(n,%)**			
Missing	66(27.62)	44(27.85)	22(27.16)
High school	89(37.24)	62(39.24)	27(33.33)
College school	78(32.64)	48(30.38)	30(37.04)
Graduate school	6(2.51)	4(2.53)	2(2.47)
**Monthly Income(RMB,n,%)**			
Missing	89(37.24)	52(32.91)	37(45.68)
≤ 3000	26(10.88)	14(8.86)	12(14.81)
3000–6000	58(24.27)	39(24.68)	19(23.46)
6000–10,000	34(14.23)	29(18.35)	5(6.17)
≥ 10,000	32(13.39)	24(15.19)	8(9.88)
**Family status(n,%)**			
Single	40(16.84)	26(16.46)	14(17.28)
Co-habiting	199(83.26)	132(83.54)	67(82.72)
**Employment status(n,%)**			
Employed	182(76.15)	135(85.44)	47(58.02)
Not Employed	57(23.85)	23(14.56)	34(41.98)
**Change in body weight in past 3 month(n,%)**			
Change	69(28.87)	36(22.78)	33(40.74)
No change	170(71.13)	122(77.22)	48(59.26)
**Smoke(n,%)**			
Yes	84(35.15)	76(48.10)	8(9.88)
No	155(64.85)	82(51.90)	73(90.12)
**Dietary structure(n,%)**			
Meat and vegetables mixed	192(80.33)	131(82.91)	61(75.31)
Meat based	26(10.88)	21(13.29)	5(25.93)
Vegetables based	21(8.79)	6(3.80)	15(18.52)
**Breakfast frequency (per week, n,%)**			
≥ 5 time	173(72.38)	127(80.38)	46(56.79)
3–4 time	35(14.64)	18(11.39)	17(20.99)
1–2 time	22(9.21)	8(5.06)	14(17.28)
None	9(3.77)	5(3.16)	4(4.94)
**Dining out frequency (per week, n,%)**			
≥ 5 time	38(15.90)	27(17.09)	11(13.58)
3–4 time	61(25.52)	51(32.28)	10(12.35)
1–2 time	128(53.56)	77(48.73)	51(62.96)
None	12(5.02)	3(1.90)	9(11.11)

### Reliability of SFFQ1 vs. SFFQ2

Table 3 presented the descriptive statistics by means (standard deviation, *SD*) of intake of 14 types of food groups and 12 types of nutrients between SFFQ1 and SFFQ2. The mean total energy intake and food intake reported in SFFQ2 were higher than that reported in SFFQ1, except the item of haslet and processed meat products. Compared with SFFQ2, the nutrient intake of vitamin B2 and calcium was slightly higher in SFFQ1. The intake of other nutrients such as carbohydrates, protein, fat, dietary fiber, vitamin C, and iron showed no significant differences between SFFQ1 and SFFQ2. The median energy-adjusted *ICC* of food groups between SFFQ2 and SFFQ1 was 0.59 (range: 0.49–0.73). The energy-adjusted *ICC* of nutrients was 0.47(range: 0.39–0.76).

**Table 3:** Reliability of food groups and nutrients intakes between the SFFQ1 and SFFQ2(n = 239)

Food groups/Nutrients	SFFQ1	SFFQ2	*ICC*	
	**Mean(SD)**	**Mean(SD)**	**Unadjusted**	**Energy-adjusted**
**Food groups(g/d)**				
Cereal	210.7(96.6)	246.4(83.8)	0.63**	0.67**
Bread and noodle	63.2(45.8)	72.4(58.6)	0.46*	0.49*
Tubers	20.6(27.2)	25.4(18.9)	0.55*	0.58*
Read meat	86.9(60.1)	97.5(53.7)	0.53**	0.57**
Poultry	52.2(42.8)	64(38.9)	0.50*	0.56*
Aquatic products	59.1(76.7)	62(72.7)	0.62*	0.53*
Haslet and processed meat products	17.5(27.6)	14.3(29.4)	0.50*	0.57*
Eggs	31.4(28.9)	40.7(19.6)	0.58**	0.69**
Dairy	92.9(110.5)	101(114.7)	0.62**	0.65**
Vegetables	238.8(144.5)	247(152)	0.71**	0.73**
Phytocomycetes	16.5(21.0)	24.1(18.8)	0.49*	0.54*
Soy products	31.9(47.9)	34.1(39.6)	0.50*	0.54*
Fruits	112.3(116.9)	121.2(89.7)	0.63**	0.67**
Nuts	13.5(23.8)	20.7(18.9)	0.51*	0.53*
**Nutrients**				
Energy(Kcal/d)	1786.3(523.4)	1820.2(220.1)	0.72**	_
Protein(g/d)	53.4(21.3)	60.3(10.8)	0.64*	0.68*
Carbohydrate(g/d)	207.2(38.4)	216.4(58.2)	0.62**	0.70**
Fat(g/d)	32.7(12.1)	35.6(9.2)	0.53*	0.69*
Dietary fiber(g/d)	49.5(54.4)	52.3(27.4)	0.34*	0.39*
Vitamin A(ugRE/d) ^a^	742.3(312.3)	772.7(603.4)	0.73*	0.76*
Vitamin B1(mg/d)	0.68(0.11)	0.74(0.31)	0.65*	0.70*
Vitamin B2(mg/d)	1.38(0.43)	1.2(0.2)	0.38*	0.40*
Vitamin C(mg/d)	115.2(41.3)	122.6(34.7)	0.67**	0.75**
Vitamin E(mg/d)	70.1(48.7)	73.4(54.7)	0.59**	0.62**
Calcium(mg/d)	567.2(162.4)	556.2(168.3)	0.52*	0.64*
Iron(mg/d)	7.1(2.2)	8.2(1.9)	0.64**	0.71**

Note: Standard deviation: *SD*, Intra-class correlation coefficient: *ICC.*

^a^: Sum of retinol, β-carotene, α-carotene, and cryptoxanthin. * *P* < 0.05. **P < 0.01.

### Validation of the SFFQ1vs. 3R24

Table 4 reported the descriptive statistics of mean (standard deviation, *SD*) intake between SFFQ1 and 3R24. Overall, the crude Pearson coefficients between SFFQ1 and 3R24 among different food groups and nutrients ranged from -0.086 to 0.93 and 0.21 to 0.71, respectively. While the energy adjusted Pearson coefficients showed higher correlation than crude coefficients, which ranged from -0.31 to 0.96 and 0.26 to 0.73, respectively. Compared with 3R24, the intake of tubers, poultry, aquatic products, eggs, dairy, phycomycetes and soy products were slightly higher in SFFQ1. The results of validity assessment of SFFQ1 *vs.* 3R24 are presented in Table 4. Phycomycetes and aquatic products showed weak correlation with the coefficient of less than 0.4. Other four food groups (such as poultry, haslet and processed meat products, tubers, soy products and nuts) showed moderate correlation with the coefficients, ranging from 0.43 to 0.58. Strong correlation with the coefficients of higher than 0.6 was observed among the following eight food groups: cereal, bread and noodles, red meat, dairy, eggs, vegetables, and fruits. The Spearman`s correlation coefficients of nutrients between SFFQ1 and3R24 ranged from 0.19 to 0.71. After adjusting the total energy intake, all correlation coefficients were increased, which ranged from 0.21 to 0.73. All correlations showed statistically significant differences between SFFQ1 and 3R24 (*P* ≤ 0.05).

**Table 4:** Validation of food groups and nutrients intakes between the SFFQ1 and 3R24 (n = 239)

Food groups/Nutrients	SFFQ1	3R24	*Rs* value	
	**Mean**(***SD)***	**Mean**(***SD)***	**Unadjusted**	**Energy-adjusted**
**Food groups(g/d)**				
Cereal	210.7(96.6)	230.2(72.0)	0.51**	0.76**
Bread and noodle	63.2(45.8)	56.4(72.7)	0.50**	0.86**
Tubers	20.6(27.2)	8.8(23.9)	0.19**	0.49**
Read meat	86.9(60.1)	110.5(57.3)	0.66**	0.72**
Poultry	52.2(42.8)	11.4(24.7)	0.16*	0.52*
Aquatic products	59.1(76.7)	12.1(28.8)	0.19*	0.32*
Haslet and processed meat products	17.5(27.6)	4.5(14.7)	0.36*	0.58*
Eggs	31.4(28.9)	13.3(23.6)	0.46**	0.78**
Dairy	92.9(110.5)	86.1(87.3)	0.57*	0.71*
Vegetables	233.9(144.5)	238.8(108.6)	0.93**	0.96**
Phytocomycetes	16.5(21.0)	2.7(13.1)	-0.086*	-0.31*
Soy products	31.9(47.9)	18.3(24.4)	0.52*	0.54*
Fruits	112.3(116.9)	156.4(130.4)	0.64**	0.65**
Nuts	13.5(23.8)	7.7(23.3)	0.32*	0.43*
**Nutrients**				
Energy(Kcal/d)	1786.3(523.4)	1923.2(356.3)	0.47*	_
Protein(g/d)	53.4(21.3)	64.3(13.6)	0.29*	0.34*
Carbohydrate(g/d)	207.2(38.4)	264.6(47.2)	0.60**	0.63**
Fat(g/d)	32.7(12.1)	56.0(19.4)	0.19*	0.21*
Dietary fiber(g/d)	49.5(54.4)	52.6(43.2)	0.21*	0.26*
Vitamin A(ugRE/d)^a^	742.3(312.3)	811.8(372.3)	0.52*	0.55*
Vitamin B1(mg/d)	0.68(0.11)	0.91(0.12)	0.38*	0.41*
Vitamin B2(mg/d)	1.38(0.43)	1.48(0.25)	0.29*	0.31*
Vitamin C(mg/d)	115.2(41.3)	131.2(32.4)	0.54*	0.62*
Vitamin E(mg/d)	70.1(48.7)	83.4(53.3)	0.71**	0.73**
Calcium(mg/d)	567.2(162.4)	541.6(167.8)	0.47*	0.52*
Iron(mg/d)	7.1(2.2)	8.2(1.7)	0.65*	0.70*

Note: Standard deviation: *SD*, Spearman correlation coeficient: *Rs*,

^a^: Sum of retinol, β-carotene, α-carotene, and cryptoxanthin. * *P* < 0.05. **P < 0.01.

## Discussion

This study examined the reliability and validity of an SFFQ that assessed the dietary intake among physical examination adults in southwest region of China. The results of usual food intake and correlation coefficients suggested that SFFQ was reproducible and well performed when compared to 3R24.

The validation of food assessment tools is essential to understand the relationships between food and nutrition-related diseases. To assess the validity, although it is always imperfect, a 24 h dietary record is used for comparing widely. In this study, the validity of SFFQ was assessed by comparing the estimates of dietary intake from a 3R24 among adults who was undergoing physical examination in southwest of China. Overall, the SFFQ demonstrated good reproducibility in estimating the intake of food groups. The crude Pearson coefficients for the correlations between daily intake of various food groups derived from the two measures ranged from -0.086 to 0.93 and the mean correlation was 0.44. The adjusted Pearson coefficients for the correlations showed improvement from 0.31 to 0.96, and the mean correlation was 0.63. The correlation coefficients in our study and other studies were found to be very similar for many food groups, showing correlations of 0.19- 0.84 [14–16].

The SFFQ and 3R24 showed some differences in their error sources, and the two methods of investigation are sufficiently independent [17]. Both questionnaires are prone to memory bias and have differences in the perception of portion sizes. The 3R24 method is based on open-ended questions, while SFFQ usually involves close-ended questions. The amount of nutritional intake can often be biased by the individuals' inability in accurately estimating the daily intake. Few other individuals showed unwillingness in acknowledging the intake of foods that might be harmful to health, such as internal organs, fatty meat, processed meat products, etc. Therefore, this SFFQ did not provide accurate and precise estimates of some nutrients (such as vitamin B, dietary fiber, protein and fat). However, for foods with low frequency of usual intake, the diet records (DRs) likely underestimates the intake of food. For example, the intake of low-frequency foods obtained by 3R24, such as tubers, poultry, aquatic products, phycomycetes, etc. in this study (in Table 4), was obviously less than those obtained by SFFQ. Although the correlation after adjusting the total energy intake was still higher than 0.3, the analysis and collection of such data seemed to be more reliable by SFFQ method. In this study, the amount of food, energy, and nutrients in repetitive SFFQ2 was increased (Table 3). This might be related to greater recall and higher attention of the subjects when the questionnaire was completed for the first time. Repeated completion of SFFQ might improve the authenticity of SFFQ. A more accurate estimation of these nutrients requires comparison of biomarkers as a reference for further study.

Fruit is the most frequently studied group in the literature and most FFQs. The correlations with regard to fruits generally range between 0.5 and 0.7, [18, 19] and this was similar to those obtained in our study (fruits: r = 0.65). Vegetables, eggs, bread and noodles, dairy and red meat showed stronger correlation (r = 0.96, 0.86, 0.78, 0.71, 0.72), which was consistent with other studies reported [13, 20]. The intake of cereal and soy products was lower than the average levels reported in China, while the intake of fruits, poultry and red meat remained higher. Other foods were similar to those obtained in the earlier studies of China health and nutrition survey (CHNS) [21]. This might be due to certain differences in the dietary and living habits of the population in southwest China The low frequency of intake of certain foods in this area might affect the results of the study. 3R24 was used to analyze, but it does not fully reflect the overall situation ofindividual`s food intake as mentioned in the previous studies [9]. Of course, increasing the days of dietary record can effectively reduce the deviation. Some scholars have collected 7 days or more longer dietary record for research [22]. But this undoubtedly increased the difficulty of the scale, which was not conducive to large-scale data collection in individuals undergoing physical examination.

There are no “gold standard” dietary investigations till date in China. Several types of instruments are used to assess both present and previous diets: the 24 h dietary recall, food-intake record, and food frequency questionnaire [19–22]. All these instruments had their own advantages and limitations. Which food intake methodology depends on the questions to be probed, the settings and participants, and the outcomes required should be investigated. A best method would be simple and quick, comprehensive and with high resolution, accurate, precise, and amenable to produce efficient and reliable data. Moreover, the main objective of the current study was to develop an easy-to-use FFQ for future epidemiological studies in China. Earlier FFQs that are used in Chinese epidemiological studies included more than 160 food items, such as CHNS [21, 22]. It would take about 30 min to finish a survey, but a lengthy questionnaire is less likely to be completed and returned [23]. The enduring participation rates of respondent effects the validation of the questionnaires [24], as longer questionnaires may cause respondent fatigue and poorer quality of gathered information. So, this might be not suitable for promotion to adults undergoing physical examination. With reference to Chinese Residents Dietary Guidelines’ selections, the FFQ was simplified, which would take 5–8 min to complete a survey, improving the operability and practicability significantly.

Generally the portion sizes are poorly estimated, and the inclusion of portion sizes in FFQs still remained controversial. For complex country such as China in terms of food, it is difficult to collect dietary information accurately using FFQ. Estimating the portion size of foods is difficult for most of the participants [25]. Some investigators have suggested the use of commonly consumed portion sizes for calculating the nutrient intake in FFQ [26]. For validating, it is important to recognize the portion size of food intake accurately. The results of this study inferred that foods with certain size or servings exhibited higher validity. For example, dairy, fruits, vegetables, and eggs, always in a certain portion size showed a higher correlation (Table 4), which was consistent with that of the previous study results [27].

It is important to mention that there are higher number of male participants and their losses (Table 2). In this study, fewer men refused to fill in the questionnaire and most of them answered the questionnaires by telephone. However, it seemed more difficult for women to complete all the questionnaires. The reason for such losses was due to the absence of 3R24, SFFQ incorrect record filling, and/or lack of informed consent. It is possible that this percentage might have influenced the results due to the final sample size.

The major strengths of this study include high participation rate, streamlined questionnaire, data collection by trained interviewers, and use of standard food map for estimating the portion size. An additional strength of this study is the normative design at the time of investigation of 3R24. The three consecutive days 24 h DRs were designed on Saturday, Monday, and Tuesday, which covered two weekdays and one weekend day, accounting for intraindividual variation between day types. Although DRs are recognized as golden standard, recording errors as well as changes in dietary habits are inevitable, especially during the weekend and holiday. The 3R24 collection method used in this study can assist in more objectively collecting the dietary data of participants on work days as well as rest days. This could help in more comprehensively comparing with the data collected by SFFQ. The SFFQ in this study has good reliability and validity in the population. It has a good application prospect in the future for analyzing the relationship between various physical examination indicators and dietary factors. It is imperative to collect health indicators and data of diet questionnaire through physical examination, conduct horizontal analysis and longitudinally monitor the health status of the population. This provides evidence-based practice for adjusting the diet structure and would promote health status of the population in the region.

The purpose of the present SFFQ is to evaluate the dietary patterns, i.e., food groups and nutrients, among adults undergoing physical health examination. The convergent validity and test–retest reliability was evaluated, but the internal consistency and discriminant validity still remained unclear. However, there are several limitations in the design of this SFFQ and validity appraisal. Firstly, it is of short length and consisted of only 14 food groups rather than single food items or dishes. Participants should classify their foods into simplified food groups, which subsequently increase the risk of misclassification. Also analysis of intake of certain special nutrients(such as anthocyanin, lycopene) might cause deviations. In addition, the reference period is only three months, which is more than one year and would be more representative of a person’s long-term dietary intake. There are seasonal variations in dietary and food intake, but the greatest seasonal variation occurs between summer and winter. Therefore, there might be a seasonal difference in the results of SFFQ and 3R24. Secondly, the repeated 24-h recalls were collected within two weeks of SFFQ1 completion. It has become difficult to predict the degree to which the divergences between the data can be attributed to questionnaire bias *vs.* differences in what individuals have actually ate during the two different time periods. While the unadjusted correlations between the items of the two questionnaires showed statistically significant differences, and some of them remained low. If the data was collected at the same time, then the unadjusted correlations might be either higher or lower. Along with all dietary assessment methods, some potential disadvantages could also be observed with regard to this SFFQ, such as recall bias, overestimation of dietary intake (particularly for rarely-consumed or healthy-perceived foods e.g., fruit and vegetables), bias of current intake and bias of pre-established food listings. Thirdly, the sample size might not reflect the mean energy intake of the population in the present study. Further study is needed for some nutrients (such as saturated and unsaturated fats, animal proteins and plant proteins, dietary fiber, vitamin B2 and vitamin B1). Finally, the problem of extrapolation within and between different food sub-groups in physical health examination adults is worth noting. Regional disparities should be taken into consideration in different areas of China. One question is that how these findings among Chinese in southwest China can be generalizable in other areas. One way to address this question is to conduct more evaluation studies to compare and validate the Chinese food cultural instruments in other areas.

## Conclusion

The reliability and validity of SFFQ questionnaire applied to the individuals undergoing physical health examination among Chinese in southwest China in this study was considered acceptable. It can be easily finished in less time when compared to FFQ, which was previously used for investigation. However, before its application in large epidemiological studies in other regions, it is necessary to re-verify the reliability and validation of SFFQ.

## Data Availability

The datasets used and/or analysed during the current study are available from the corresponding author on reasonable request.

## Abbreviations

SFFQ: Simple food frequency questionnaire
3R24: Three-day 24-h dietary record
ICC: Intra-class correlation coefficients
NTCDs: Non-transmissible chronic diseases
R24: 24 h dietary record
SD: Standard deviation
HCNS: China Health and Nutrition Survey

## References

[1] Rippin HL, Hutchinson J, Evans CEL, et al. National nutrition surveys in Europe: a review on the current status in the 53 countries of the WHO European region. Food Nutr Res. 2018;62:10.29219/fnr.v62.1362. 10.29219/fnr.v62.1362 PMC5917420 29720930

[2] Luana SM, Bruna KH, Camilla CPE (2017). Food Consumption According to the Days of the Week – National Food Survey, 2008–2009. Rev SaudePublica.

[3] Rawiwan S, Celine H, Ella H (2019). Comparison of phytosterol intake from FFQ with repeated 24HDRs of the Adventist Health Study-2 calibration sub-study. Br J Nutr.

[4] Liisa K, Henna V, Carola R (2019). Parents’Reports of Preschoolers’Diets: Relative Validity of a Food Frequency Questionnaire and Dietary Patterns. Nutrients.

[5] Khaoula EK, Vanessa GL, Mohamed K (2018). Adaptation and validation of a food frequency questionnaire (FFQ) to assess dietary intake in Moroccan adults. Nutr J.

[6] Karianne S, Hege BH, Beate Ø, et al. Evaluation of a short Food Frequency Questionnaire to assess cardiovascular disease-related diet and lifestyle factors, Food Nutr Res. 2018; 62: 10.29219/fnr.v62.1370. 10.29219/fnr.v62.1370 PMC5917418 29720928

[7] Synnøve N, Inger A, Marian Kd, et al. Validation and reproducibility of a new iodine specific food frequency questionnaire for assessing iodine intake in Norwegian pregnant women. Nutr J. 2019;18(1):62. 10.1186/s12937-019-0489-4 PMC6821006 31665021

[8] Allehdan SS, Tayyem RF, Agraib LM (2019). Relative Validity and Reproducibility of a Food Frequency Questionnaire to Assess Food Group Intake in Pregnant Jordanian Women. J AcadNutr Diet.

[9] Giovannelli J, Dallongeville J, Wagner A (2014). Validation of a short, qualitative food frequency questionnaire in French adults participating in the MONA LISA-NUT study 2005–2007. J AcadNutr Diet.

[10] Wang ZH, Penny GL, Anna MS (2017). Sociodemographic Disparity in the Diet Quality Transition among Chinese adults, 1991 to 2011. Eur J ClinNutr.

[11] Lee H, Kang M, Song WO, et al. Gender analysis in the development and validation of FFQ: A systematic review. Br J Nutr. 2016;115(4):666–71. 10.1017/S0007114515004717 26652249

[12] Prentice RL, Mossavar-Rahmani Y, Huang Y, et al. Evaluation and comparison of food records, recalls, and frequencies for energy and protein assessment by using recovery biomarkers. Am J Epidemiol. 2011;174(5):591–603. 10.1093/aje/kwr140 PMC3202154 21765003

[13] Yoko H, Kaoru K, Kanha S, et al. Development and validation of a food frequency questionnaire (FFQ) for assessing dietary macronutrients and calcium intake in Cambodian school-aged children. Nutr J. 2019;18(1):11. 10.1186/s12937-019-0437-3 PMC6385409 30791913

[14] Geeta A, Gerda KP, Therese AO (2014). The reliability of an adolescent dietary pattern identified using reduced-rank regression: comparison of a FFQ and 3 d food record. Br J Nutr.

[15] Nithya N, Clare W, Sharna S (2016). Development of a Semi-Quantitative Food Frequency Questionnaire to Assess the Dietary Intake of a Multi-Ethnic Urban Asian Population. Nutrients.

[16] Ikuko K, Mauro S, Junko I (2018). The Validity and Reproducibility of Dietary Non-enzymatic Antioxidant Capacity Estimated by Self-administered Food Frequency Questionnaires. J Epidemiol.

[17] Yatiman NH, Lee CA, Fendy Y (2019). Validity and Reliability of a Food Frequency Questionnaire (FFQ) to Assess Dietary Intake of Preschool Children. Int J Environ Res Public Health.

[18] Huang MC, Lin KD, Chen HJ (2018). Validity of a Short Food Frequency Questionnaire Assessing Macronutrient and Fiber Intakes in Patients of Han Chinese Descent with Type 2 Diabetes. Int J Environ Res Public Health.

[19] Tatiana SC, Augusto CFDM, Tara RU (2019). The Validity of Children`s Fruit and Vegetable Intake Using Plasma Vitamins A, C, and E: The SAYCARE Study. Nutrients.

[20] Peter JH, Sylvia HL, Shilpa NBDSF, et al. Associations of dietary, lifestyle, and sociodemographic factors with iron status in Chinese adults: a cross-sectional study in the China Health and Nutrition Survey. Am J Clin Nutr. 2017;105(2):503–12. 10.3945/ajcn.116.136861 PMC6546221 28031193

[21] Fang AP, Li KJ, Li H (2017). Low Habitual Dietary Calcium and Linear Growth from Adolescence to Young Adulthood: results from the China Health and Nutrition Survey. Sci Rep.

[22] Monnerie B, Tavoularis LG, Guelinckx I (2015). A cross-over study comparing an online versus a paper 7-day food record: focus on total water intake data and participant`s perception of the records. Eur J Nutr.

[23] Keshteli A, Esmaillzadeh A, Rajaie S (2014). A Dish-based Semi-quantitative Food Frequency Questionnaire for Assessment of Dietary Intakes in Epidemiologic Studies in Iran Design and Development. Int J Prev Med.

[24] Watanabe D, Nanri H, Yoshida T, et al. Validation of energy and nutrition intake in Japanese elderly individuals estimated based on a short food frequency questionnaire compared against a 7-day dietary record: the Kyoto-Kameoka study. Nutrients. 2019;11:688. 10.3390/nu11030688 PMC6471352 30909514

[25] Kaija N, Liisa K, Henna V, et al. Accuracy in the estimation of children’s food portion sizes against a food picture book by parents and early educators. J NutrSci. 2018;7:e35. 10.1017/jns.2018.26 PMC6313402 30627432

[26] Christine H, Lubowa A (2019). Simple methods to obtain food listing and portion size distribution estimates for use in semi-quantitative dietary assessment methods. PLoS ONE.

[27] Liu D, JU La H, Yang ZY, et al. Food Frequency Questionnaire for Chinese Children Aged 12–17 Years: Validity and Reliability. Biomed Environ Sci, 2019;32(7):486–95. 10.3967/bes2019.066 31331433

